# Time-dependent integrated tissue and circulating biomarker dynamics of spexin and progranulin in an experimental myocardial infarction model

**DOI:** 10.1007/s00418-026-02471-z

**Published:** 2026-03-29

**Authors:** Sercan Kaya, Tuba Yalçin

**Affiliations:** Vocational Higher School of Healthcare Studies, Batman University, Main Campus, Health Services Vocational School, Room 212, Kültür Neighborhood, Batman, Turkey

**Keywords:** Myocardial infarction, Isoproterenol, Biomarkers, Spexin, Progranulin

## Abstract

Inflammation, oxidative stress and apoptosis, in particular, play critical roles in the pathophysiology of myocardial infarction (MI). Isoproterenol (ISO) is a frequently used agent in experimental models to induce MI. This study aimed to determine the temporal changes in Spexin (SPX) and Progranulin (PRG) levels in an ISO-induced experimental MI model. The study consisted of control and ISO-treated groups (ISO-1, ISO-2, ISO-4, ISO-6 and ISO-24). In the ISO-induced MI model, serum CK-MB and troponin (I-T) levels increased, confirming cardiac damage. However, increases in oxidative parameters and decreases in antioxidant enzyme levels indicated cardiac oxidative stress. Furthermore, the time-dependent increase in inflammatory cell infiltration on histopathological examinations, paralleled by the increase in proinflammatory cytokines, confirmed the cardiac inflammatory response. Despite a decrease in cardiac antiapoptotic markers, the increase in proapoptotic marker levels reflected the temporal change in the apoptotic process induced by ISO administration. ISO administration caused SPX levels in serum and cardiac tissue to increase at 4 h and decrease at 24 h. The dynamic fluctuations in SPX levels may be related to adaptation to the stress responses and cardiac energy metabolism. ISO administration increased PRG levels in serum and cardiac tissue at 6 and 24 h. This increase suggests that PRG may contribute to a role particularly in inflammation and cardiac remodeling processes. In conclusion, the cardiac oxidative stress, inflammation and apoptosis induced by ISO administration further validated the experimental MI model. Changes in SPX and PRG levels in the ISO-induced MI model demonstrate their promise as cardiac potential markers.

**Graphical Abstract:**

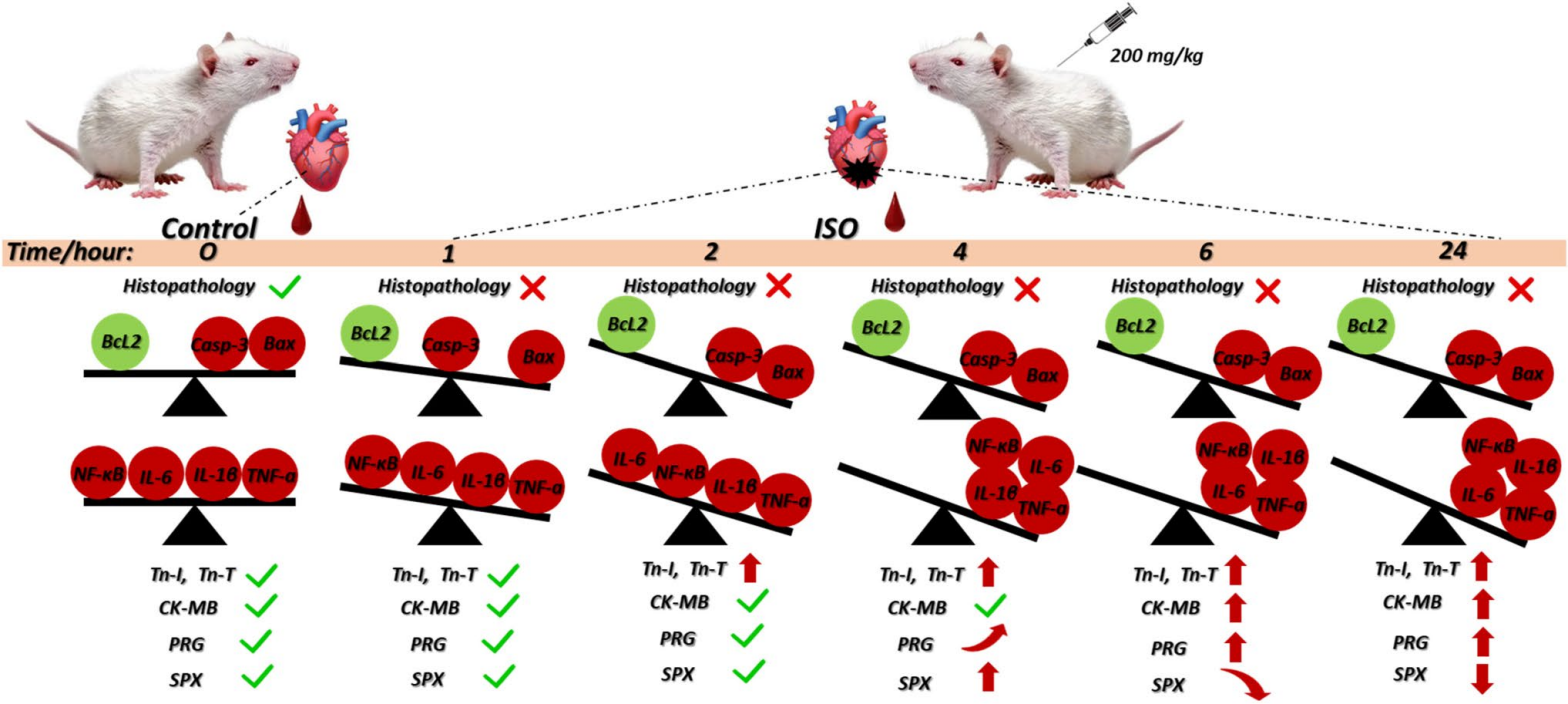

## Introduction

Myocardial infarction (MI), a leading cause of death worldwide, continues to be a serious health problem (Ahmad et al. 2022). The pathophysiology of MI involves disruption of the homeostatic balance between myocardial oxygen supply and demand, resulting in cardiomyocyte death (Kaya and Yalcin 2023). Many factors, including excessive production of inflammatory cytokines and reactive oxygen species (ROS), play a role in the development and progression of MI (Althunibat et al. 2022). Experimental designs are crucial for determining the detailed mechanisms of MI and evaluating potential markers and new cardioprotective strategies (Martin et al. 2022). Isoproterenol (ISO) is a β-adrenergic agonist frequently used in experimental models to induce MI (Khan et al. 2018). Excessive ROS production due to autooxidation of catecholamines underlies ISO-induced cardiac damage, and this is considered a major cause of MI (Říha et al. 2014). ISO-induced oxidative stress in cardiac tissue triggers inflammatory and apoptotic processes, leading to cardiac dysfunction (Senoner and Dicht 2019).

Spexin (SPX), discovered through bioinformatic analyses, is a 14-amino acid neuropeptide (Mirabeau et al. 2007). SPX is encoded by the Ch12:orf39 gene, is expressed in many tissues and is secreted into the systemic circulation (Porzionato et al. 2010; Kaya et al. 2023a). Studies investigating receptor-ligand interaction mechanisms have reported that SPX has high affinity for galanin receptors GAL2 and GAL3 (Yalcin and Kaya 2025). In this context, a previous study demonstrated that GAL1-3 receptor agonists have cardioprotective effects (Studneva et al. 2019). In support of this, a recent study reported that SPX reduces susceptibility to atrial fibrillation via GAL2 receptors (Li et al. 2024). Furthermore, SPX pretreatment was reported to preserve mitochondrial balance and cardiomyocyte energy homeostasis under hypoxic conditions (Liu et al. 2020). Clinical studies have linked SPX to biomarkers associated with glucose metabolism and cardiovascular disease (Kumar et al. 2018; Khadir et al. 2020). Taken together, these findings suggest that SPX may serve as a potential biomarker in the early stages of cardiovascular disease.

Following myocardial necrosis due to interruption of coronary artery blood flow, a rapid inflammatory response is initiated to facilitate the migration of macrophages and monocytes into the infarcted myocardial tissue (Nahrendorf and Swirski 2013). Progranulin (PRG) is a glycoprotein containing 593 amino acids (Bateman and Bennett 1998). PRG, which plays a role in tissue repair and modulation of the immune response, is particularly expressed in immune cells such as macrophages and neutrophils (Schmitz et al. 2020). Studies have reported that PRG exhibits a protective effect against ischemic damage in brain and kidney tissues (Egashira et al. 2013; Zhou et al. 2015). Furthermore, PRG administration provided a protective effect against cardiac damage after MI in mice and rabbits (Sasaki et al. 2020). PRG has also been reported to play a role in regulating macrophage polarization and function (Sasaki et al. 2023). However, the exact role of endogenous PRG in cardiac remodeling after MI is unknown.

The aim of this study was to determine cardiac damage in an ISO-induced experimental MI model and to examine the course of SPX and PRG levels in serum and heart tissues at 1, 2, 4, 6 and 24 h time points following ISO administration.

## Materials and methods

### Experimental design

All experimental procedures were performed in accordance with the Arrive guidelines, and approval was obtained from the Firat University Animal Experiments Local Ethics Committee (dated May 27, 2024, no. 24456) before starting the study. Forty-two 8- to 10-week-old female Sprague-Dawley rats were used in the experiment. The animals were housed at 22–25 °C with a 12-h light/dark cycle. Standard rat chow was used for feeding, and food and water were provided ad libitum. The animals were randomly divided into six groups of seven rats each.

Control (*n* = 7): Rats in this group received no treatment during the experiment. Control group rats were decapitated along with rats in the ISO-24 group.

ISO-1 (*n* = 7): To induce experimental MI, 200 mg/kg ISO (isoproterenol hydrochloride, Sigma-Aldrich, St. Louis, MO, USA) was dissolved in normal saline and administered as a single subcutaneous dose. Rats were decapitated 1 h after ISO injection.

ISO-2 (*n* = 7): Experimental MI was induced, and rats were decapitated 2 h after ISO injection.

ISO-4 (*n* = 7): Experimental MI was induced, and rats were decapitated 4 h after ISO injection.

ISO-6 (*n* = 7): Experimental MI was induced, and rats were decapitated 6 h after ISO injection.

ISO-24 (*n* = 7): Experimental MI was induced, and rats were decapitated 24 h after ISO injection.

No adverse events or animal deaths were reported during the experiment. Following completion of the experimental procedures, intracardiac blood samples were collected from rats under anesthesia at the specified time points. Anesthesia was induced with xylazine (10 mg/kg) and ketamine (75 mg/kg), followed by decapitation. The collected blood and heart tissues were used for biochemical, histopathological and immunohistochemical analyses.

### Histopathological evaluations

Heart tissue samples obtained at the end of the experiment were fixed in 10% formalin solution for histopathological examination, then embedded in paraffin blocks using routine histological procedures; 5-µm-thick sections were cut from the paraffin blocks using a microtome. For general histopathological evaluations, heart tissue sections were stained with hematoxylin-eosin (H&E) and examined using a light microscope (× 20 objective, Air, NA 0.50, DM2500 LED, Leica Microsystems, Wetzlar, Germany) images were photographed (MC170 HD, Leica, Wetzlar, Germany). To evaluate histopathological changes in heart tissue sections, 10 randomly selected non-overlapping fields were examined under × 20 magnification on sections prepared separately for each rat, and histopathological findings were scored (0: absent, 1: slight, 2: moderate, 3: severe) (Kaya et al. 2023b). Histopathological criteria evaluated included myofibril loss, intracytoplasmic vacuolization, mononuclear cell infiltration, erythrocyte extravasation and edema.

### Immunohistochemical evaluations

The 5-µm-thick sections taken from paraffin blocks and mounted on polysine-coated slides were deparaffinized and cleared before being passed through a series of decreasing alcohol concentrations. The avidin-biotin peroxidase complex (ABC) method was used to detect SPX (Boster/A04088–1, Pleasanton, CA, USA) and PRG (DF7997/Affinity, Camarillo, CA, USA) immunoreactivities in heart tissues. AEC chromogen was then used as the enzyme substrate. Immunohistochemical evaluation was based on the extent (0–100%) and severity (0: absent, 0.5: very slight, 1: slight, 2: moderate, 3: intense) of immunoreactivity. Prevalence values ​​were graded as 0.1 (< 25%), 0.4 (26–50%), 0.6 (51–75%) and 0.9 (76–100%). The histoscore table was created with the formula immunoreactivity = prevalence × severity (Yalcin et al. 2024). In addition, TNF-α (AF7014/Affinity, Camarillo, CA, USA), IL-1β (Santa Cruz/sc-1251, Dallas, TX, USA) and NF-κB (AF5006/Affinity, Camarillo, CA, USA) immunohistochemical stainings were applied to evaluate the pro-inflammatory response in heart tissue sections. Apoptotic processes were evaluated by examining the immunoreactivities of anti-apoptotic BcL-2 (201r.5304/SunRed, Shanghai Sunred Biological Technology Co., Ltd, China), pro-apoptotic Bax (sc-7480/Santa Cruz, Dallas, TX, USA) and Casp-3 (AF6311/Affinity, Camarillo, CA, USA).

The primary antibodies used in this study (SPX, PRG, TNF-α, IL-1β, NF-κB, BcL-2, Bax and Casp-3) were validated for specificity by the manufacturers, and specificity was further confirmed by positive/negative tissue controls in accordance with the “Antibody Validation Policy” in the Springer Submission Guidelines. In addition, all antibody catalog numbers and supplier addresses are provided to ensure the reproducibility of the experiments.

### Biochemical assessments

Commercial ELISA (enzyme-linked immunosorbent assay) kits were used to determine circulating levels of SPX (BT Lab./E2567Ra) and PRG (BT Lab./E1222Ra) in serum samples. Additionally, levels of routine cardiac biomarkers troponin I (Tn-I; BT Lab./E0305Ra), Tn-T (BT Lab./E0306Ra) and CK-MB (BT Lab./E0311Ra) were also measured using ELISA. Heart tissues were homogenized in 10% phosphate buffer solution and centrifuged (at 4 °C, 10 min, 5000 × g) to obtain supernatants. Rat-specific ELISA kits were commercially available for quantitative measurement of SPX and PRG levels, and supernatants were analyzed according to the manufacturer's instructions. In addition, the oxidant/antioxidant parameters malondialdehyde (MDA; BT Lab./E0156Ra), catalase (CAT; BT Lab./E0869Ra), superoxide dismutase (SOD; BT Lab./E0168Ra) and glutathione (GSH; BT Lab./EA0113Ra) were determined in heart tissue homogenate using ELISA. Inflammatory markers TNF-α (BT Lab./E0764Ra) and IL-6 (BT Lab./E0135Ra) were also measured in heart tissue homogenate using ELISA. All ELISA kits used in this study were obtained from Bioassay Technology Laboratory (BT Lab, Shanghai Korain Biotech Co., Ltd, Shanghai 200,090, China).

### Statistical analyses

**Fig. 1:**
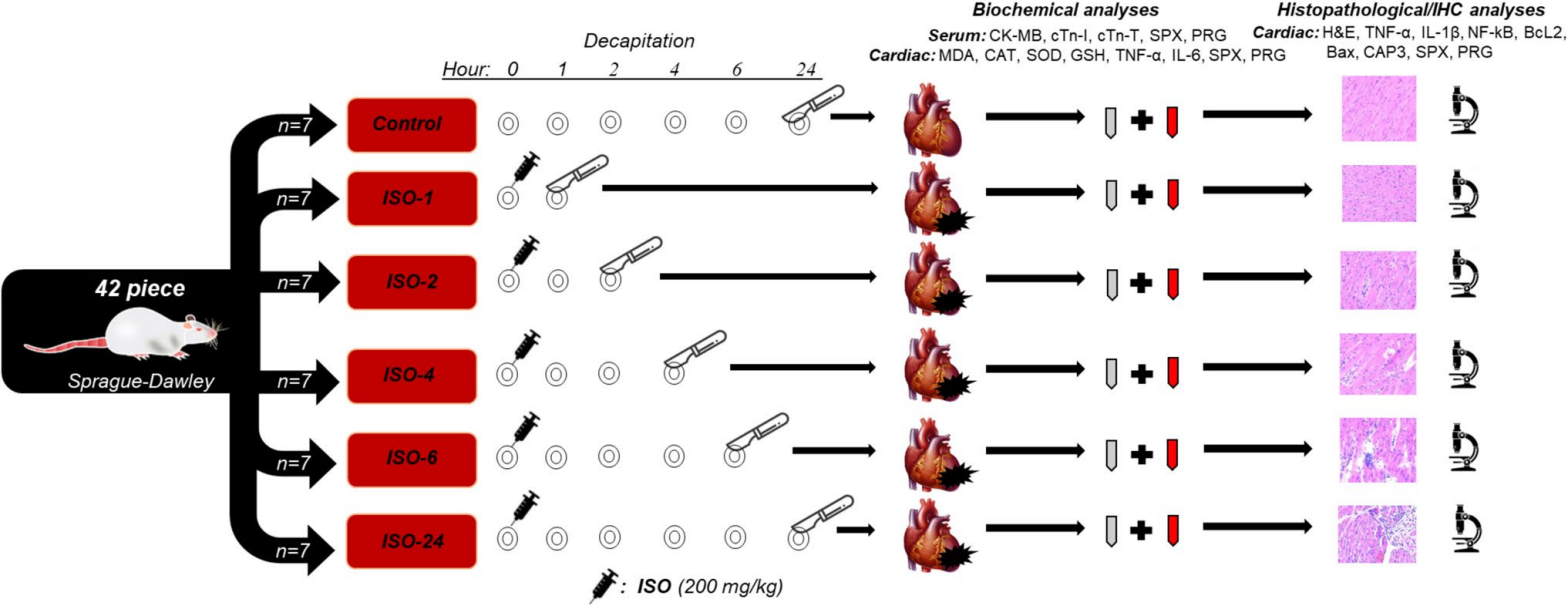
Experimental design

Statistical analyses of the data obtained in the study were performed using SPSS 22.0 software. The Shapiro-Wilk test was used to assess the conformity of the data to a normal distribution. One-way ANOVA and post hoc Tukey tests were applied for normally distributed data. Data are presented as mean ± standard deviation. For data that did not show a normal distribution, pairwise comparisons were made using the Kruskal-Wallis test, followed by the Mann-Whitney U test. Data are presented as medians (minimum-maximum). Statistical significance was accepted as *p* < 0.05. Graphical presentations of the data were created using GraphPad Prism 9.3 software (Fig. 1).

## Results

### Oxidant/antioxidant parameter levels in the ISO-induced MI model

**Fig. 2:**
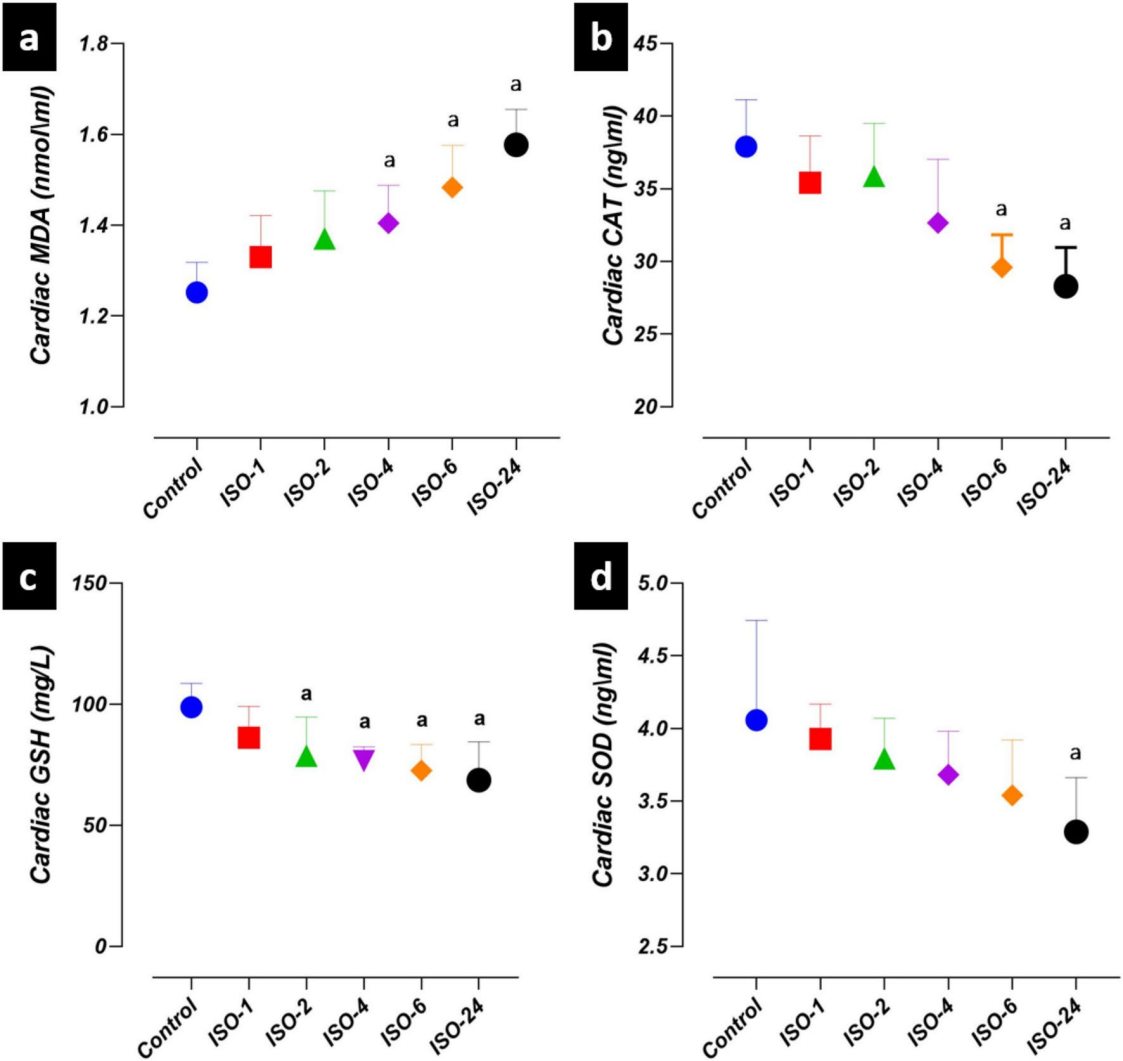
Oxidant/antioxidant parameter levels in the ISO-induced MI model at different time points. When comparing the time points following ISO administration in heart tissue with the control group, MDA levels increased at 4 h, CAT levels decreased at 6 h, GSH levels decreased at 2 h, and SOD levels decreased at 24 h. **a** Heart tissue malondialdehyde (MDA) level. **b** Heart tissue catalase (CAT) level. **c** Heart tissue glutathione (GSH) level. **d** Heart tissue superoxide dismutase (SOD) level

Significant differences were found among the levels of MDA, CAT, GSH and SOD at different time points of control and ISO administration. MDA levels in heart tissue increased at 4 h following ISO administration and were higher in the ISO-4, ISO-6 and ISO-24 groups compared with the control group (*p* < 0.05). CAT levels in heart tissue decreased at 6 h following ISO administration and were lower in the ISO-6 and ISO-24 groups than in the control group (*p* < 0.05). GSH levels in heart tissue decreased at 2 h following ISO administration and were lower in the ISO-2, ISO-4, ISO-6 and ISO-24 groups than in the control group (*p* < 0.05). However, SOD levels in heart tissue decreased at 24 h following ISO administration (ISO-24) compared with the control group (*p* < 0.05) (Fig. 2).

### Troponin (I-T) and CK-MB levels in the ISO-induced MI model

**Fig. 3:**
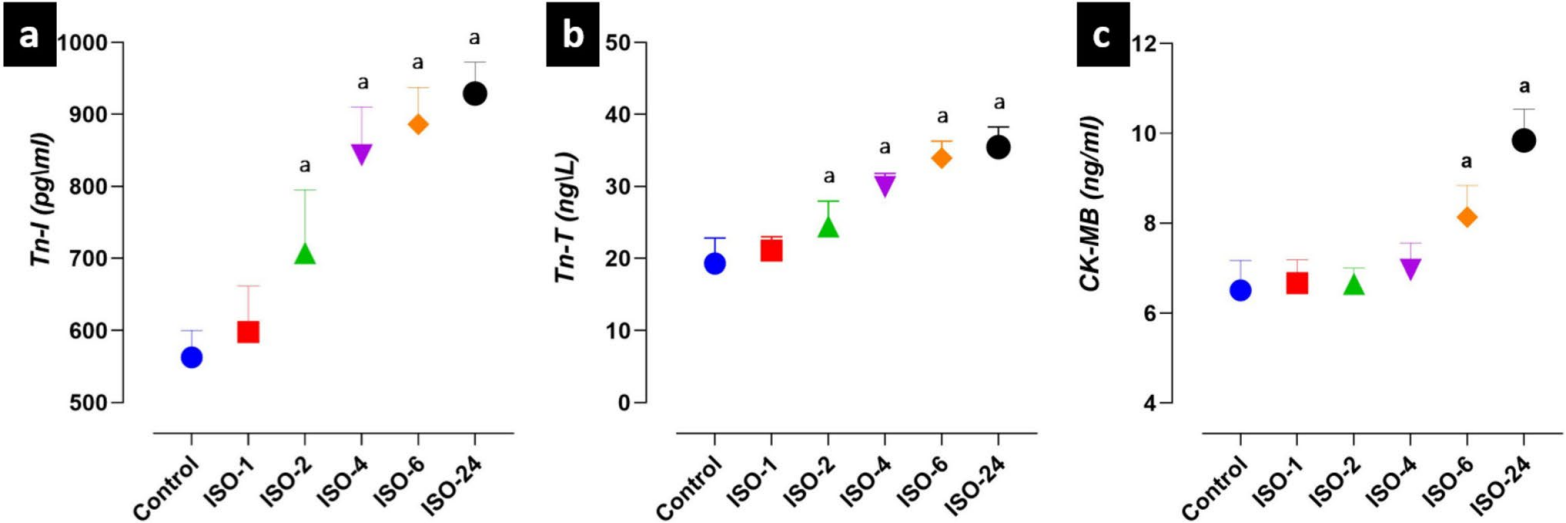
Troponin (I-T) and CK-MB levels at different time points in the ISO-induced MI model. When the time points following ISO administration were compared with the control group, serum Tn-I and Tn-T levels increased at 2 h, and CK-MB levels increased at 6 h. **a** Serum troponin-I level. **b** Serum troponin-T level. **c** Serum creatine kinase-MB (CK-MB) level

Significant differences were found among Tn-I, Tn-T and CK-MB levels at different time points in the control and ISO administration groups. Serum Tn-I and Tn-T levels increased at 2 h following ISO administration and were higher in the ISO-2, ISO-4, ISO-6 and ISO-24 groups than in the control group (*p* < 0.05). Serum CK-MB levels increased at 6 h following ISO administration and were higher in the ISO-6 and ISO-24 groups than in the control group (*p* < 0.05) (Fig. 3).

### Histopathological evaluation of heart tissue in the ISO-induced MI model

**Fig. 4:**
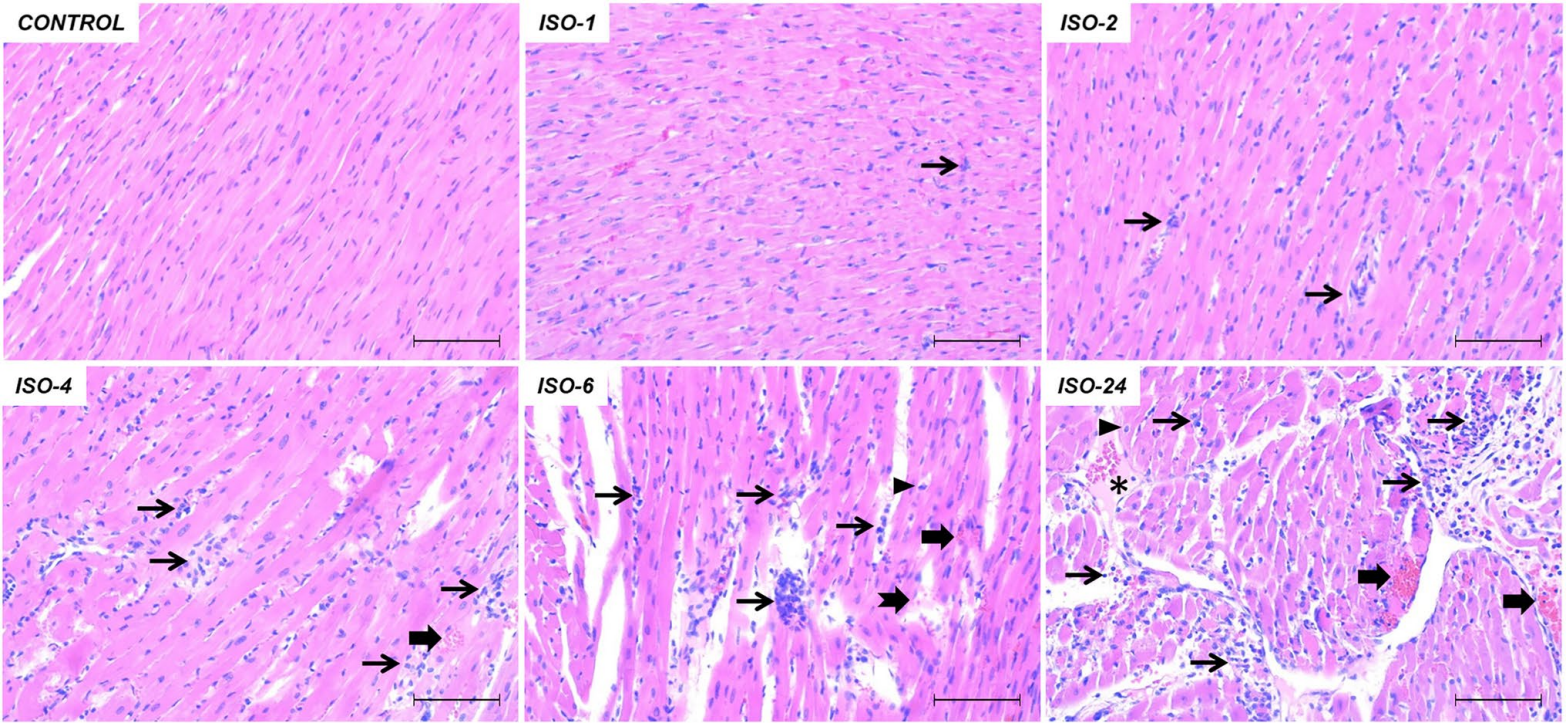
Histopathological evaluation of heart tissue in the ISO-induced MI model. The heart tissue in the control group had a normal histological structure. Following ISO administration, mononuclear cell infiltration was observed at 1 h, erythrocyte extravasation at 4 h, intracytoplasmic vacuolization and myofibril loss at 6 h, and cardiac edema at 24 h. These histopathological changes increased over time. Thin arrow: Mononuclear cell infiltration. Thick arrow: erythrocyte extravasation. Arrowhead: intracytoplasmic vacuolization. Notched arrow: myofibril loss. Star: edema. Hematoxylin & eosin staining. Scale bar 100 µm. ISO: isoproterenol

The heart tissues in the control group had a normal histological structure. ISO administration was found to cause histopathological changes in the heart tissue at various time points. Mononuclear cell infiltration, which increased at 1 h following ISO administration, continued to increase at subsequent time points. However, histopathological changes such as erythrocyte extravasation, intracytoplasmic vacuolization and myofibril loss were commonly observed at 4 and 6 h. Histopathological changes, including cardiac edema, were significantly increased at 24 h (*p* < 0.05) (Table 1, Fig. 4).

**Table 1:** Results of histopathological evaluation of heart tissue in the ISO-induced MI model

	Control Med (min–max)	ISO-1 Med (min–max)	ISO-2 Med (min–max)	ISO-4 Med (min–max)	ISO-6 Med (min–max)	ISO-24 Med (min–max)	p*
Mononuclear cell infiltration	0.00 (0.00–0.10)	0.20 (0.10–0.40)^a^	0.40 (0.20–0.60)^a^	0.80 (0.60–1.20)^a^	1.30 (0.90–1.60)^a^	1.90 (1.70–2.50)^a^	< 0.01
Erythrocyte extravasation	0.00 (0.00–0.10)	0.20 (0.10–0.40)^a^	0.40 (0.30–0.50)^a^	0.60 (0.50–0.90)^a^	1.20 (0.70–1.40)^a^	1.60 (1.30–1.90)^a^	< 0.01
Edema	0.00 (0.00–0.00)	0.00 (0.00–0.10)	0.10 (0.00–0.20)^a^	0.20 (0.00–0.30)^a^	0.30 (0.20–0.50)^a^	1.40 (1.20–1.90)^a^	< 0.01
Intracytoplasmic vacuolization	0.00 (0.00–0.10)	0.20 (0.00–0.30)^a^	0.30 (0.20–0.50)^a^	0.50 (0.40–0.70)^a^	0.90 (0.70–1.30)^a^	1.50 (1.10–1.70)^a^	< 0.01
Myofibril loss	0.00 (0.00–0.00)	0.00 (0.00–0.10)	1.00 (0.00–0.20)^a^	0.50 (0.30–0.70)^a^	0.90 (0.60–1.30)^a^	1.30 (0.80–1.60)^a^	< 0.01

Data are presented as median (minimum–maximum)

^a^Compared with the control group (*p* < 0.05)

p* Kruskal-Wallis

### Heart tissue inflammatory marker levels in the ISO-induced MI model

**Fig. 5:**
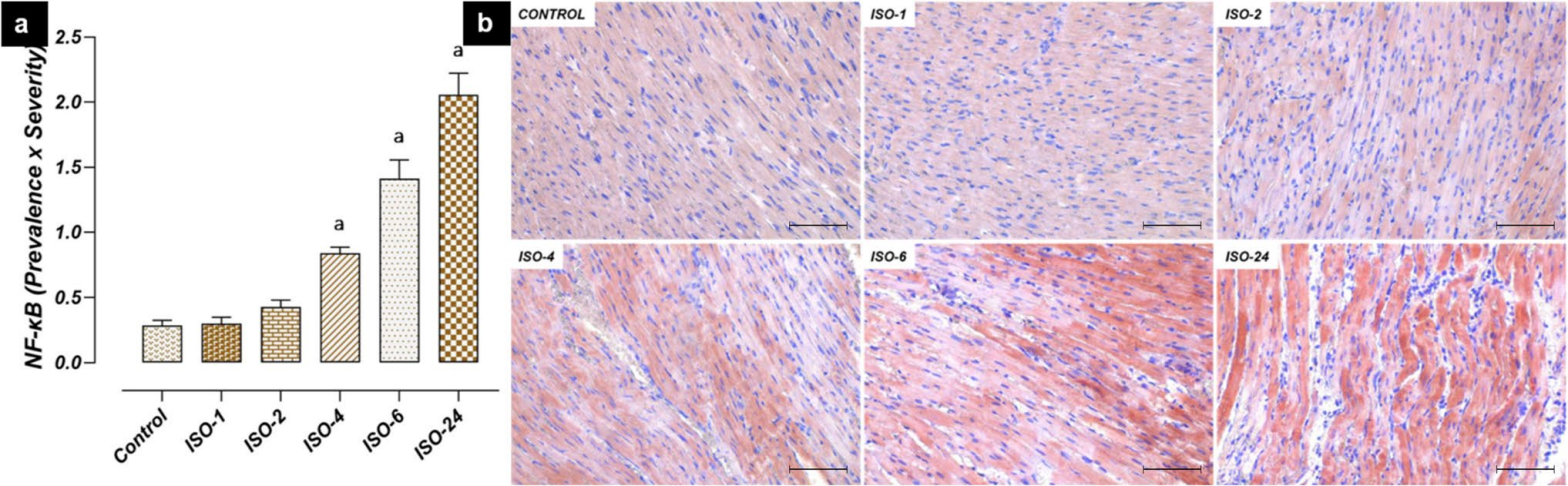
NF-κB immunoreactivity in heart tissue in the ISO-induced MI model. NF-κB immunoreactivity in heart tissue increased at 4 h following ISO administration compared with the control group and persisted at subsequent time points. **a** NF-κB IHC evaluation graph. **b** NF-κB IHC-staining microphotographs. Scale bar: 100 µm. *IHC* immunohistochemistry, *ISO* isoproterenol, *NF-κB* nuclear factor kappa B

Significant differences were detected between the control and ISO administration time points in NF-κB, IL-1β, IL-6 and TNF-α levels. NF-κB immunoreactivity in heart tissue increased at 4 h following ISO administration and was found to be higher in the ISO-4, ISO-6 and ISO-24 groups than in the control group (*p* < 0.05) (Fig. 5).

**Fig. 6:**
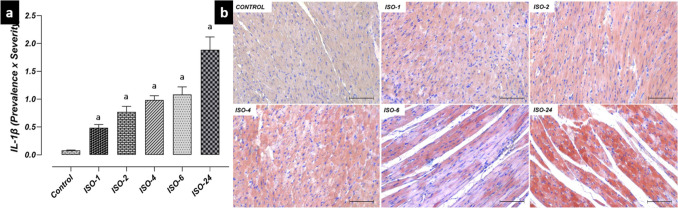
Heart tissue IL-1β immunoreactivity in the ISO-induced MI model. IL-1β immunoreactivity in heart tissue increased at 1 h following ISO administration compared with the control group and continued at subsequent time points. **a** IL-1β IHC evaluation graph. **(b)** IL-1β IHC-staining microphotographs. Scale bar: 100 µm. *IHC* immunohistochemical, *ISO* isoproterenol, *IL-1β* interleukin-1β

**Fig. 7:**
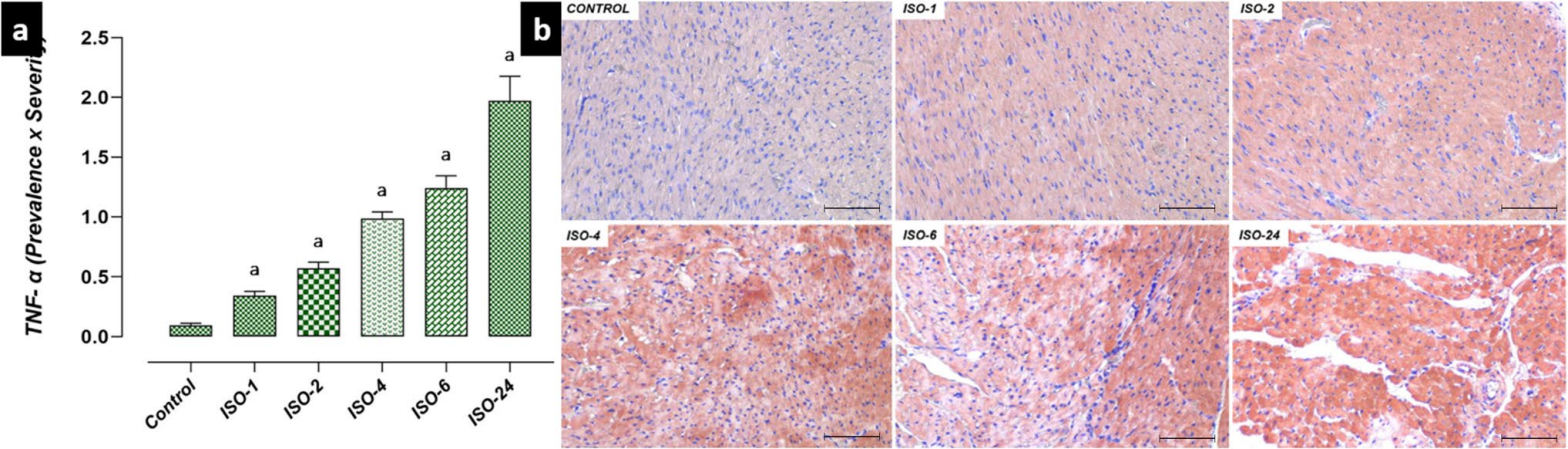
Heart tissue TNF-α immunoreactivity in the ISO-induced MI model. TNF-α immunoreactivity in heart tissue increased at 1 h following ISO administration compared with the control group and continued at subsequent time points. **a** TNF-α IHC evaluation graph. **b** TNF-α IHC-staining microphotographs. Scale bar: 100 µm. *IHC* immunohistochemical, *ISO* isoproterenol, *TNF-α* tumor necrosis factor-alpha

Similarly, IL-1β and TNF-α immunoreactivities in heart tissue increased at 1 h following ISO administration and were found to be higher in the ISO-1, ISO-2, ISO-4, ISO-6 and ISO-24 groups compared with the control group (*p* < 0.05) (Fig. 6, Fig. 7).

**Fig. 8:**
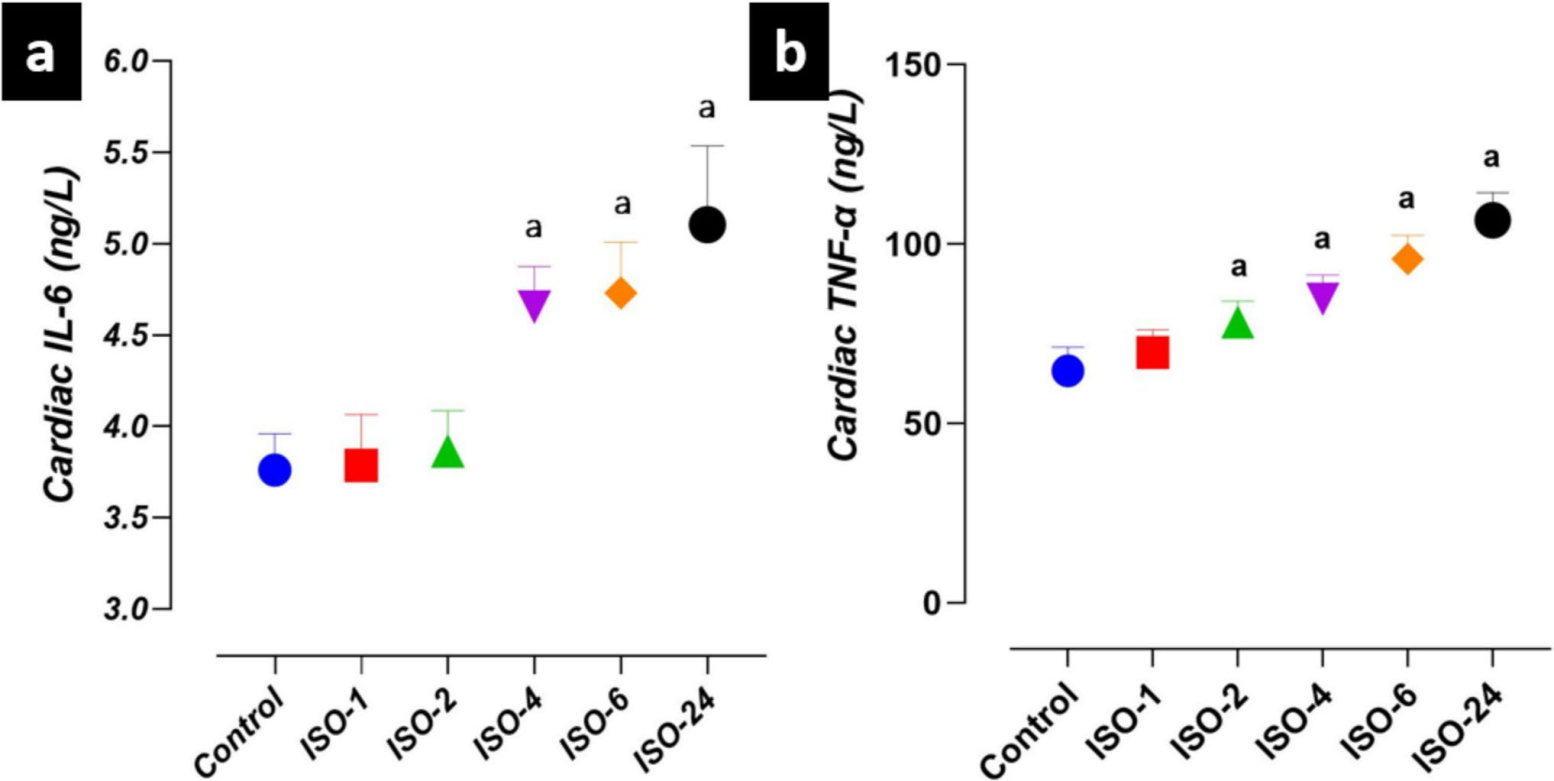
IL-6 and TNF-α levels in heart tissue homogenate in the ISO-induced MI model. Compared with the control group, TNF-α levels in heart tissue increased at 2 h and 4 h following ISO administration, and this increase continued at subsequent time points. **a** Cardiac tissue IL-6 levels. **b** Cardiac tissue TNF-α levels. *ISO* isoproterenol, *IL-6* interleukin-6, *TNF-α* tumor necrosis factor-alpha

However, IL-6 levels in heart tissue homogenate increased at 4 h following ISO administration and were higher in the ISO-4, ISO-6 and ISO-24 groups than in the control group (*p* < 0.05). Similarly, TNF-α levels in heart tissue homogenate increased at 2 h following ISO administration and were higher in the ISO-2, ISO-4, ISO-6 and ISO-24 groups compared with the control group (*p* < 0.05) (Fig. 8).

### Heart tissue apoptotic marker levels in the ISO-induced MI model

**Fig. 9:**
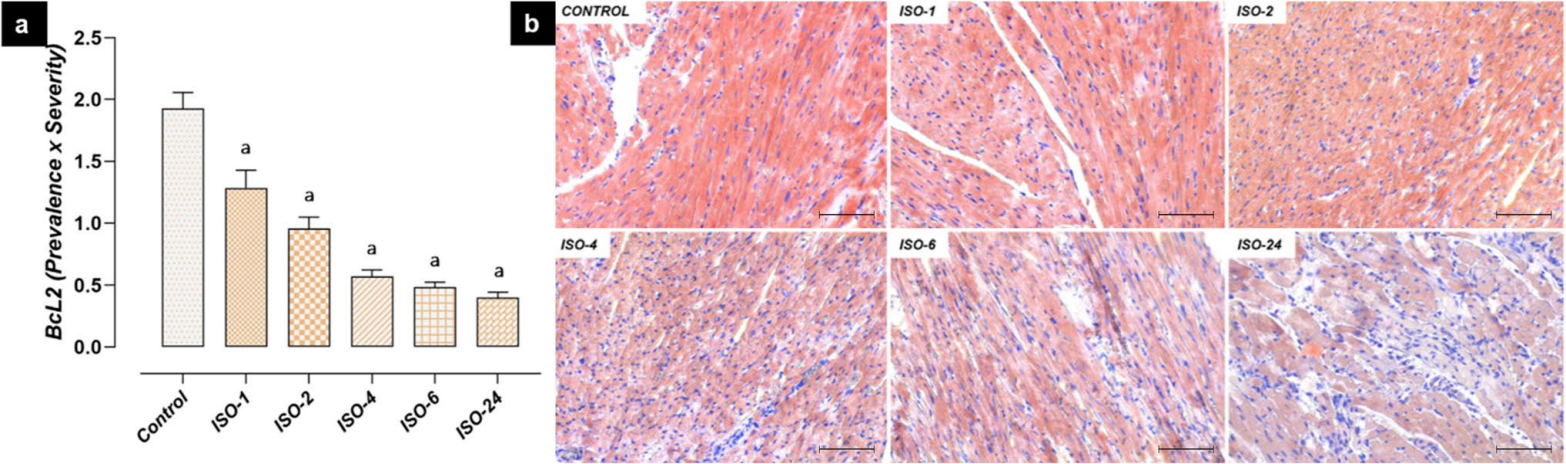
Heart tissue BcL-2 immunoreactivity in the ISO-induced MI model. BcL-2 immunoreactivity in heart tissue decreased at 1 h following ISO administration compared with the control group, and this decrease continued at subsequent time points. **a** BcL-2 IHC evaluation graph. **b** BcL-2 IHC-staining microphotographs. Scale bar: 100 µm. IHC: immunohistochemical, ISO: isoproterenol

Significant differences were found between the levels of anti-apoptotic BcL-2 and pro-apoptotic Bax and Casp-3 at different time points between control and ISO administration. BcL-2 immunoreactivity in heart tissue decreased at 1 h following ISO administration and was lower in the ISO-1, ISO-2, ISO-4, ISO-6 and ISO-24 groups than in the control group (*p* < 0.05) (Fig. 9).

**Fig. 10:**
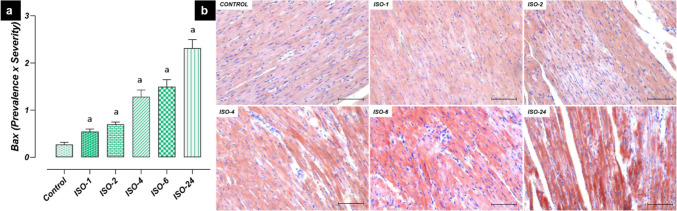
Bax immunoreactivity in heart tissue in the ISO-induced MI model. Bax immunoreactivity in heart tissue increased at 1 h following ISO administration compared with the control group, and this increase continued at subsequent time points. **a** Bax IHC evaluation graph. **b** Bax IHC-staining microphotographs. Scale bar: 100 µm. *IHC* immunohistochemical, *ISO* isoproterenol

However, Bax immunoreactivity in heart tissue increased at 1 h following ISO administration, and an increase was found in the ISO-1, ISO-2, ISO-4, ISO-6 and ISO-24 groups compared with the control group (*p* < 0.05) (Fig. 10).

**Fig. 11:**
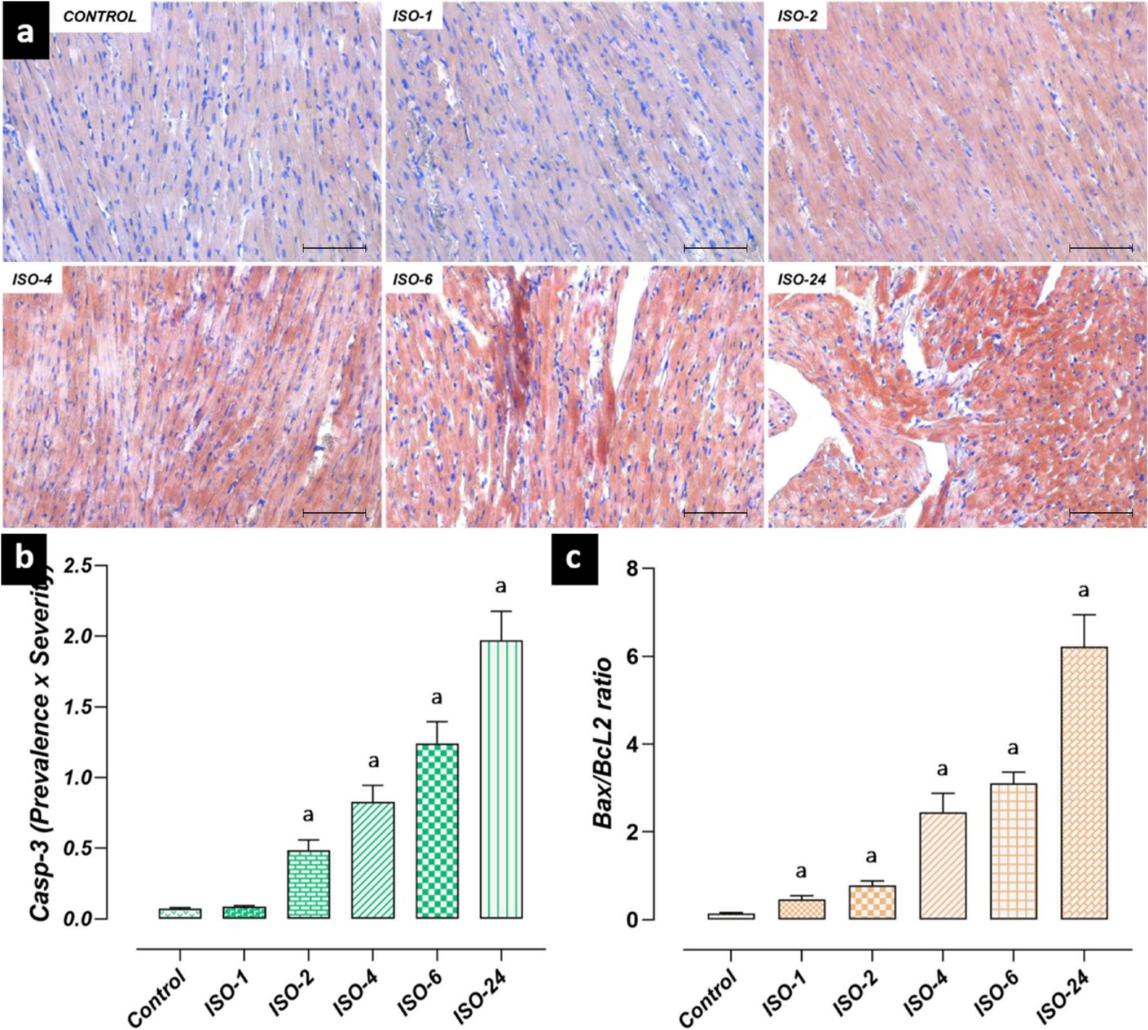
Heart tissue Casp-3 immunoreactivity and Bax/BcL-2 ratio in the ISO-induced MI model. Casp-3 immunoreactivity in heart tissue increased at 2 h following ISO administration compared with the control group, and this increase continued at subsequent time points. Similarly, the Bax/BcL-2 ratio increased at 1 h following ISO administration compared with the control group, and this increase continued at subsequent time points. **a** Casp-3 IHC-staining microphotographs. Scale bar: 100 µm. **b** Casp-3 IHC evaluation graph. **c** Bax/BcL-2 ratio graph. *IHC* immunohistochemical, *ISO* isoproterenol

However, Casp-3 immunoreactivity in heart tissue increased at 2 h following ISO administration, and an increase was found in the ISO-2, ISO-4, ISO-6 and ISO-24 groups compared with the control group (*p* < 0.05). Additionally, it was determined that the Bax/BcL-2 ratio in heart tissue increased at 1 h following ISO administration compared with the control group, and this increase continued at subsequent time points (*p* < 0.05) (Fig. 11).

### SPX Levels in the ISO-induced MI model

**Fig. 12:**
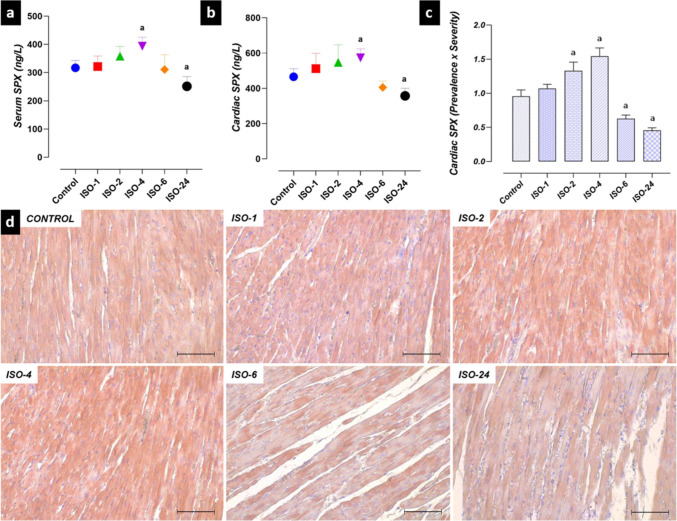
SPX levels in the ISO-induced MI model. SPX serum and heart homogenate levels increased at 4 h and decreased at 24 h following ISO administration compared with the control group. Similarly, SPX immunoreactivity was higher at 2 and 4 h and decreased at 6 and 24 h following ISO administration compared with the control group. **a** Serum SPX level graph. **b** Heart homogenate SPX level graph. **c** SPX IHC evaluation graph. **d** SPX IHC staining microphotographs Scale bar: 100 µm. *IHC* immunohistochemical, *ISO* isoproterenol, *SPX* Spexin

Significant differences were found between SPX levels at different time points after ISO administration and control. SPX levels in serum and heart tissue homogenate increased at 4 h and decreased at 24 h following ISO administration compared with the control group. Similarly, SPX immunoreactivity was found to increase in the ISO-2 and ISO-4 groups and decrease in the ISO-6 and ISO-24 groups compared with the control group (*p* < 0.05) (Fig. 12).

### PRG levels in the ISO-induced MI model

**Fig. 13:**
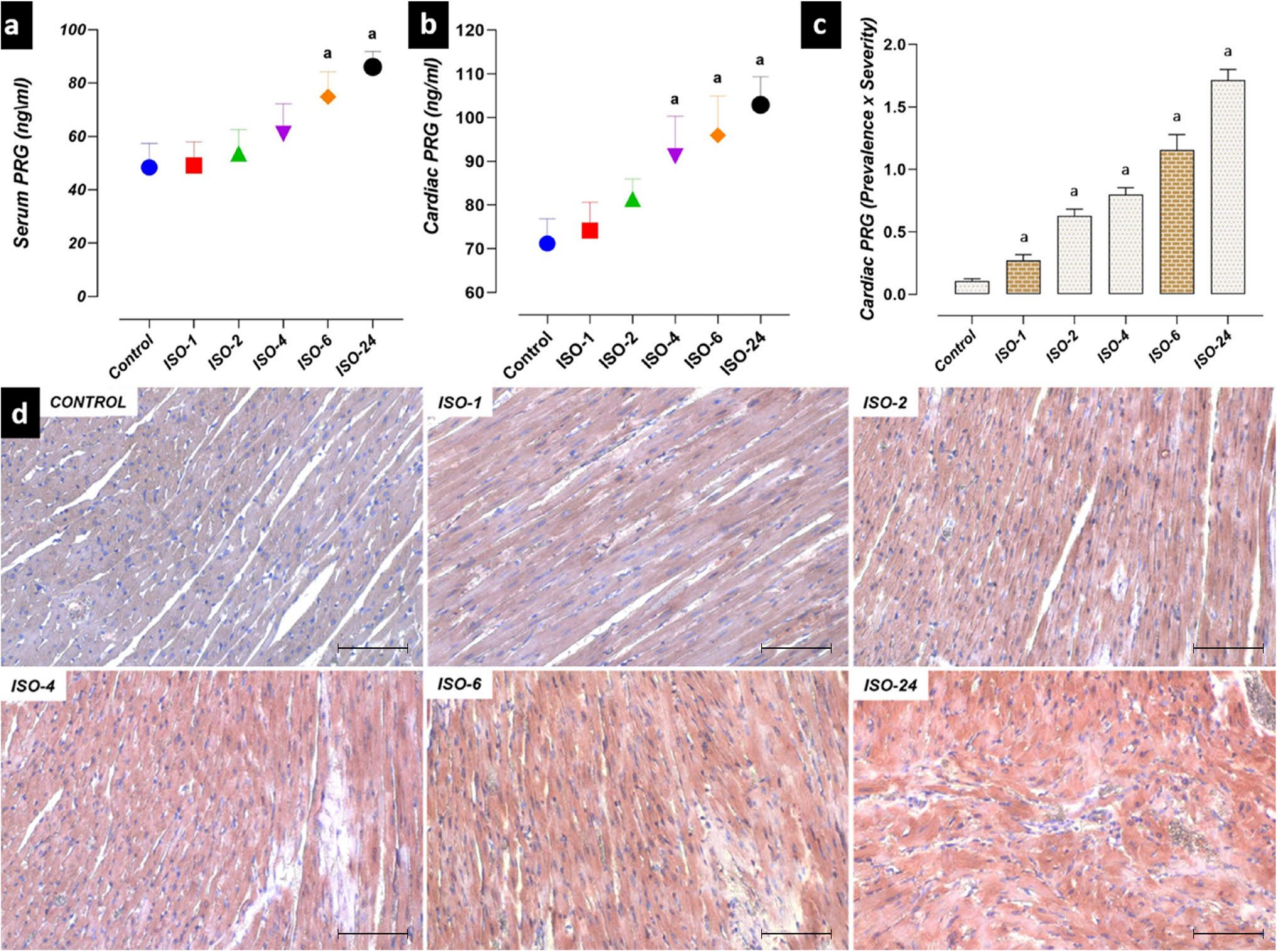
PRG levels in the ISO-induced MI model. PRG levels in heart homogenate and serum were increased at 4 and 6 h following ISO administration, respectively, compared with the control group. Similarly, PRG immunoreactivity increased at 1 h following ISO administration, compared with the control group, and increases in PRG levels were detected at subsequent time points. **a** Serum PRG level graph. **b** Heart homogenate PRG level graph. **c** PRG IHC evaluation graph. **d** PRG IHC staining microphotographs Scale bar: 100 µm. *IHC* immunohistochemical, *ISO* isoproterenol, *PRG* progranulin

Significant differences were found between PRG levels at different time points of control and ISO administration. PRG levels in the heart tissue homogenate increased at 4 h following ISO administration compared with the control group, while serum PRG levels increased at 6 h. Similarly, PRG immunoreactivity increased at 1 h following ISO administration compared with the control group. PRG levels continued to increase after the time point at which they increased (*p* < 0.05) (Fig. 13).

## Discussion

This current study further confirmed that the ISO-induced MI model reliably captures the fundamental biochemical and histopathological changes of MI. Furthermore, it demonstrated that PRG and SPX, in addition to clinically used cardiac markers (TnI, TnT and CK-MB), hold promise as potential biomarkers for inflammatory and metabolic processes in myocardial injury. This study highlights the role of fluctuating SPX levels in the stress response and cardiac energy metabolism during MI, while the observed increase in Prg levels highlights its potential role in cardiac inflammation and repair.

ISO is a nonselective β-adrenergic agonist (Pandi et al. 2022). ISO-induced cardiotoxicity causes MI-like damage. Although several mechanisms have been proposed for myocardial damage resulting from ISO administration, excessive ROS production during the auto-oxidation of catecholamines is considered one of the most important causes (Song et al. 2020). Oxidized ISO suppresses the antioxidant system in the cell through lipid peroxidation by producing quinone (Anajirih et al. 2024). Studies have shown that myocardial hypoxia and ischemia cause oxidative stress and trigger excessive ROS production (Zarkovic 2020; Wei et al. 2021). It has also been reported that ROS production resulting from mitochondrial oxidative phosphorylation can trigger oxidative stress (Ray et al. 2012). MDA levels, a product of lipid peroxidation, and antioxidant parameters (such as CAT, SOD and GSH) are markers indicating oxidative damage (Martínez-Noguera et al. 2021). In this context, the current study measured MDA, CAT, SOD and GSH levels to determine the level of oxidative stress in ISO-induced cardiac tissue. The study findings showed that ISO administration increased MDA levels in cardiac tissue while decreasing antioxidant parameters such as CAT, SOD and GSH. Consistent with these results, a study reported decreased GSH, SOD, CAT and glutathione peroxidase (GPx) levels in the ISO-induced MI model (Yin et al. 2022). MDA, which disrupts the mitochondrial membrane potential, can lead to cellular damage by affecting cellular proteins and DNA integrity (Shanab et al. 2023). Increased lipid peroxidation and MDA disrupt the membrane permeability and integrity of cardiomyocytes, increasing the levels of cardiac enzymes (such as CK-MB) in circulation (Liu et al. 2018). A study reported that MDA accumulation in heart tissue is positively correlated with CK-MB levels (Anajirih et al. 2024).

Serum levels of myocardial enzymes are crucial in determining the severity of MI. Under physiological conditions, circulating levels of cardiac enzymes found in the cardiomyocyte cytoplasm are quite low. However, when cardiomyocytes are damaged, membrane integrity is disrupted, myocardial enzymes are released into the circulation, and serum levels increase (Liu et al. 2017; Wu et al. 2024). In this current study, serum Tn-I, Tn-T and CK-MB levels increased following ISO administration. These results were consistent with previous studies reporting increased cTn-I, cTn-T and CK-MB levels in the ISO-induced MI model (Yin et al. 2022). Furthermore, the increase in circulating cardiac markers in the current study and biochemical results confirming cardiac oxidative stress are consistent with our histopathological findings demonstrating degenerative changes in cardiomyocytes.

The strong correlation between oxidative stress and inflammation suggests that inflammation is an additional mechanism underlying ISO-induced cardiotoxicity (Jain et al. 2018). Excessive ROS production triggers a cellular signaling cascade that increases the levels of proinflammatory cytokines and mediators, resulting in inflammation (Shanab et al. 2023). Proinflammatory cytokine amplification and the inflammatory response are associated with maladaptive cardiac remodeling and increased mortality (Coggins and Rosenzweig 2012). However, activation of the transcription factor NF-κB in cardiac injury can increase the expression of proinflammatory cytokines such as IL-1β and TNF-α, leading to inflammation and fibrosis (de Castro et al. 2018). NF-κB is a redox-regulated transcription factor that is inactive under physiological conditions but recruits to the nucleus when triggered (Habotta et al. 2023). Subsequently, it promotes proinflammatory mediators, including TNF-α, by increasing the activation of adhesion molecules via IL-6, and causes the accumulation of mononuclear cells at the site of inflammation (Shanab et al. 2024). In this study, it was found that the levels of NF-κB, TNF-α, IL-1β and IL-6 were increased in heart tissue in the ISO-induced MI model. Furthermore, this study confirmed the strong relationship between oxidative stress and inflammation in ISO-induced cardiac injury. Moreover, histopathological evaluation of the heart tissue showed significant inflammatory cell infiltration. These findings are consistent with previous studies reporting increased levels of inflammation-related markers in the MI model (Asiwe et al. 2023; Anajirih et al. 2024).

Apoptosis is cell death that occurs through the initiation of specific cellular programs controlled by complex regulatory mechanisms (Sun et al. 2024). Excessive accumulation of ROS accelerates the apoptosis process in cardiomyocytes, impairing heart function and contributing to the progression of cardiovascular diseases (Zarkovic 2020; Wei et al. 2021). The apoptosis process is regulated by two cytoplasmic proteins: anti-apoptotic BcL-2 and pro-apoptotic Bax. During apoptosis, Bax protein triggers mitochondrial intrinsic signaling pathways, leading to cell death (Kulsoom et al. 2018). On the other hand, BcL-2 can prevent apoptosis by suppressing Bax protein (de Castro et al. 2018). The levels of these proteins can be considered biological indicators for predicting apoptosis (Yue et al. 2020). A decrease in the BcL-2/Bax ratio activates the Casp-3 cascade, leading to the degradation of poly(ADP-ribose) polymerase and apoptosis (Zhuang et al. 2021). In this study, anti-apoptotic BcL-2 levels were decreased in the ISO-induced MI model, while pro-apoptotic Bax levels were increased. Furthermore, increased Bax/BcL-2 ratios and Casp-3 levels were also observed. Similarly, a recent study reported that a decrease in the BcL-2/Bax ratio was an indicator of cardiomyocyte apoptosis and that this activated Casp-3, initiating the apoptotic process (Cui et al. 2024).

Hypoxia is a potential trigger for heart diseases such as MI. One study showed that SPX may improve hypoxia-induced energy metabolism disorders and mitochondrial dysfunction by regulating fatty acid metabolism in cardiomyocytes, increasing ATP production and reducing mitochondrial ROS production. The same study reported decreased SPX levels in cardiomyocytes of mice exposed to hypoxia (Liu et al. 2020). Conversely, another study reported increased SPX expression in peripheral chemoreception after exposure to hyperoxia, suggesting that this is due to SPX's sensitivity to oxygen concentration (Porzionato et al. 2012). In this current study, SPX levels in an experimental MI model were found to increase at 4 h following ISO administration but then decreased, with SPX levels being significantly lower at 24 h. Considering that glucose and fatty acid metabolism disorders contribute to cardiac damage in experimental hypoxia models (Tao et al. 2019), we believe that SPX may play a role in adaptation to the pathophysiological conditions that emerge in the early stages of ISO administration and, therefore, its increase. The decrease in SPX levels observed with the increase in oxidative stress, inflammation and apoptotic markers partially supports this notion. In support of this notion, a study in mouse heart tissues showed that pretreatment with SPX limited ROS production during hypoxia, supported fatty acid metabolism, protected against mitochondrial damage and increased ATP levels in cardiomyocytes (Liu et al. 2020). Furthermore, SPX has been reported to play a role in inflammation in addition to its metabolic role (Kumar et al. 2018). In this current study, the increase in pro-inflammatory markers such as NF-κB, TNF-α, IL-1β and IL-6 ultimately reduced SPX levels in the ISO-induced MI model. A similar study showed that SPX reduced the expression of pro-inflammatory markers such as IL-1, IL-6 and TNF in obese rats (Gambaro et al. 2020).

High expression of PRG has been observed in macrophages and vascular smooth muscle cells in atherosclerotic arteries in the cardiovascular system (Kojima et al. 2009). Furthermore, PRG plays a role in atherosclerosis, being one of the main causes of infarction and myocardial ischemia (Al Masri and Al Ha 2015). One study showed that PRG mRNA expression is strongly increased in cardiac tissue after myocardial ischemia-reperfusion (I/R) injury (Alyahya et al. 2019). PRG expression is increased in immune cells, including neutrophils and macrophages, during ischemic conditions (Kanazawa et al. 2015). Studies have reported that PRG is abundantly expressed in infiltrating macrophages at the border zones of MI (Sasaki et al. 2020; 2023). Another study reported that serum PRG levels were significantly increased in patients with acute MI and that PRG was an independent risk factor in acute MI patients (Zhou et al. 2021). In this current study, PRG levels increased at 6 h following ISO administration and maintained an upward trend at 24 h. Supporting this result, a previous study reported that PRG was expressed in cardiac tissue, particularly in the border zones of the infarcted myocardium and in infiltrating macrophages. The same study demonstrated that PRG deficiency negatively affected macrophage infiltration, fibrosis and cardiac remodeling after MI (Sasaki et al. 2023). Another study reported that PRG gradually increased in cardiac tissue 1, 3 and 7 days after MI. PRG exhibited cardioprotective effects by promoting reparative macrophage infiltration in MI (Sasaki et al. 2020). Furthermore, accumulating evidence suggests that PRG plays an important role in reducing myocardial fibrosis and promoting cardiac repair after I/R injury (Tian et al. 2016; Sasaki et al. 2020). While increased PRG levels in MI were initially thought to be a marker of poor cardiovascular risk, recent evidence suggests a cardioprotective role for PRG in MI and I/R injury.

A significant limitation of this study is that cardiac PRG and SPX levels were not further confirmed by analyses such as Western blot. More importantly, while the study revealed dynamic changes in PRG and SPX levels during MI, it did not provide information about PRG and SPX levels during post-MI cardiac remodeling. Future studies could focus on the role of potential cardiac markers such as PRG and SPX in cardiac remodeling processes during and after MI.

In conclusion, this study confirmed once again that the ISO-induced MI model reliably reflects myocardial oxidative stress, inflammation and apoptosis. The study results demonstrated that dynamic changes in PRG and SPX levels are associated with cardiac injury, metabolic response and repair mechanisms. PRG and SPX may be considered promising biomarkers for understanding the pathophysiology of MI and identifying potential diagnostic or therapeutic targets.

## Data Availability

The datasets used and/or analyzed during the current study are available from the corresponding author upon reasonable request.

## References

[CR1] Ahmad T, Khan T, Tabassum T, Alqahtani YS, Mahnashi MH, Alyami BA et al (2022) Juglone from walnut produces cardioprotective effects against isoproterenol-induced myocardial injury in SD rats. Curr Issues Mol Biol 44:3180–3193 35877444 10.3390/cimb44070220 PMC9319353

[CR2] Al Masri A, Al HA (2015) The adipokine, progranulin (PGRN), is cardio-protective in myocardial ischemia reperfusion injury in the rat. Atherosclerosis 241:e207–e208

[CR3] Althunibat OY, Abduh MS, Abukhalil MH, Aladaileh SH, Hanieh H, Mahmoud AM (2022) Umbelliferone prevents isoproterenol-induced myocardial injury by upregulating Nrf2/HO-1 signaling, and attenuating oxidative stress, inflammation, and cell death in rats. Biomed Pharmacother 149:112900 35378502 10.1016/j.biopha.2022.112900

[CR4] Alyahya AM, Al-Masri A, Hersi A, El Eter E, Husain S, Lateef R et al (2019) The effects of progranulin in a rat model of acute myocardial ischemia/reperfusion are mediated by activation of the PI3K/Akt signaling pathway. Med Sci Monit Basic Res 25:229–237 31695019 10.12659/MSMBR.916258 PMC6859783

[CR5] Anajirih N, Abdeen A, Taher ES, Abdelkader A, Abd-Ellatieff HA, Gewaily MS et al (2024) Alchemilla vulgaris modulates isoproterenol-induced cardiotoxicity: Interplay of oxidative stress, inflammation, autophagy, and apoptosis. Front Pharmacol 15:1394557 39170697 10.3389/fphar.2024.1394557 PMC11335554

[CR6] Asiwe JN, Ben-Azu B, Yovwin GD, Ehebha SE, Igben VJO, Ahama EE et al (2023) Enhancements of Bcl-2/mTOR/ERK1/2 activities by antioxidant mechanisms confer cardioprotection on *Ginkgo biloba* supplement against isoprenaline-induced myocardial infarction in rats. Pharmacol Res Mod Chin Med 8:100293

[CR7] Bateman A, Bennett HP (1998) Granulins: the structure and function of an emerging family of growth factors. J Endocrinol 158:145–151 9771457 10.1677/joe.0.1580145

[CR8] Coggins M, Rosenzweig A (2012) The fire within: cardiac inflammatory signaling in health and disease. Circ Res 110:116–125 22223209 10.1161/CIRCRESAHA.111.243196

[CR9] Cui Y, Wu J, Wang Y, Li D, Zhang F, Jin X et al (2024) Protective effects of ginsenoside F2 on isoproterenol-induced myocardial infarction by activating the Nrf2/HO-1 and PI3K/Akt signaling pathways. Phytomedicine 129:155637 38669969 10.1016/j.phymed.2024.155637

[CR10] de Castro AL, Fernandes RO, Ortiz VD, Campos C, Bonetto JH, Fernandes TRG et al (2018) Thyroid hormones decrease the proinflammatory TLR4/NF-κB pathway and improve functional parameters of the left ventricle of infarcted rats. Mol Cell Endocrinol 461:132–142 28888669 10.1016/j.mce.2017.09.003

[CR11] Egashira Y, Suzuki Y, Azuma Y et al (2013) The growth factor progranulin attenuates neuronal injury induced by cerebral ischemia-reperfusion through the suppression of neutrophil recruitment. J Neuroinflammation 10:2–13 23972823 10.1186/1742-2094-10-105 PMC3765381

[CR12] Gambaro SE, Zubiría MG, Giordano AP, Portales AE, Alzamendi A, Rumbo M, Giovambattista A (2020) Spexin improves adipose tissue inflammation and macrophage recruitment in obese mice. Biochim Biophys Acta Mol Cell Biol Lipids 1865:158700 32201217 10.1016/j.bbalip.2020.158700

[CR13] Habotta OA, Abdeen A, El-Hanafy AA, Yassin N, Elgameel D, Ibrahim SF et al (2023) Sesquiterpene nootkatone counteracted the melamine-induced neurotoxicity via repressing oxidative stress, inflammatory, and apoptotic trajectories. Biomed Pharmacother 165:115133 37454594 10.1016/j.biopha.2023.115133

[CR14] Jain PG, Mahajan UB, Shinde SD, Surana SJ (2018) Cardioprotective role of ferulic acid against isoproterenol-induced cardiac toxicity. Mol Biol Rep 45:1357–1365 30105550 10.1007/s11033-018-4297-2

[CR15] Kanazawa M et al (2015) Multiple therapeutic effects of progranulin on experimental acute ischaemic stroke. Brain 138:1932–1948 25838514 10.1093/brain/awv079

[CR16] Kaya S, Yalçin T (2023) In an experimental myocardial infarction model, L-arginine pre-intervention may exert cardioprotective effects by regulating OTULIN levels and mitochondrial dynamics. Cell Stress Chaperones 28:811–820 37644219 10.1007/s12192-023-01373-6 PMC10746646

[CR17] Kaya S, Yalçin T, Boydak M, Dönmez HH (2023a) Protective effect of N-acetylcysteine against aluminum-induced kidney tissue damage in rats. Biol Trace Elem Res 201(4):1806–1815 35553365 10.1007/s12011-022-03276-6

[CR18] Kaya S, Yalçin T, Kuloğlu T (2023b) Resveratrol may reduce oxidative stress and apoptosis in doxorubicin-induced cardiotoxicity by regulating meteorin-like and TRPM2 levels. Comp Clin Pathol 32:393–404

[CR19] Khadir A, Kavalakatt S, Madhu D, Devarajan S, Abubaker J, Al-Mulla F et al (2020) Spexin as an indicator of beneficial effects of exercise in human obesity and diabetes. Sci Rep 10:10635 32606431 10.1038/s41598-020-67624-z PMC7327065

[CR20] Khan V, Sharma S, Bhandari U, Ali SM, Haque SE (2018) Raspberry ketone protects against isoproterenol-induced myocardial infarction in rats. Life Sci 194:205–212 29225109 10.1016/j.lfs.2017.12.013

[CR21] Kojima Y, Ono K, Inoue K, Takagi Y, Kikuta K, Nishimura M et al (2009) Progranulin expression in advanced human atherosclerotic plaque. Atherosclerosis 206:102–108 19321167 10.1016/j.atherosclerosis.2009.02.017

[CR22] Kulsoom B, Shamsi TS, Afsar NA, Memon Z, Ahmed N, Hasnain SN (2018) Bax, Bcl-2, and Bax/Bcl-2 as prognostic markers in acute myeloid leukemia: Are we ready for Bcl-2-directed therapy? Cancer Manag Res 10:403–416 29535553 10.2147/CMAR.S154608 PMC5841349

[CR23] Kumar S, Hossain MJ, Javed A, Kullo IJ, Balagopal PB (2018) Relationship of circulating spexin with markers of cardiovascular disease: a pilot study in adolescents with obesity. Pediatr Obes 13:374–380 29045048 10.1111/ijpo.12249 PMC5906205

[CR24] Li D, Liu Y, Li C, Zhou Z, Gao K, Bao H et al (2024) Spexin diminishes atrial fibrillation vulnerability by acting on galanin receptor 2. Circulation 150:111–127 38726666 10.1161/CIRCULATIONAHA.123.067517

[CR25] Liu ZY, Hu SP, Ji QR, Yang HB, Zhou DH, Wu FF (2017) Sevoflurane pretreatment inhibits the myocardial apoptosis caused by hypoxia reoxygenation through AMPK pathway: an experimental study. Asian Pac J Trop Med 10:148–151 28237479 10.1016/j.apjtm.2017.01.006

[CR26] Liu J, Chen L, Lu H (2018) Cardioprotective effect of salvianolic acid B against isoproterenol-induced inflammation and histological changes in a cardiotoxicity rat model. Trop J Pharm Res 17:2189–2197

[CR27] Liu Y, Sun L, Zheng L, Su M, Liu H, Wei Y et al (2020) Spexin protects cardiomyocytes from hypoxia-induced metabolic and mitochondrial dysfunction. Naunyn-Schmiedebergs Arch Pharmacol 393:25–33 31396649 10.1007/s00210-019-01708-0

[CR28] Martin TP, MacDonald EA, Elbassioni AAM, O’Toole D, Zaeri AAI, Nicklin SA et al (2022) Preclinical models of myocardial infarction: from mechanism to translation. Br J Pharmacol 179:770–791 34131903 10.1111/bph.15595

[CR29] Martínez-Noguera FJ, Alcaraz PE, Ortolano-Ríos R, Dufour SP, Marín-Pagán C (2021) Differences between professional and amateur cyclists in endogenous antioxidant system profile. Antioxidants (Basel) 10:282 33673363 10.3390/antiox10020282 PMC7918641

[CR30] Mirabeau O, Perlas E, Severini C, Audero E, Gascuel O, Possenti R et al (2007) Identification of novel peptide hormones in the human proteome by hidden Markov model screening. Genome Res 17:320–327 17284679 10.1101/gr.5755407 PMC1800923

[CR31] Nahrendorf M, Swirski FK (2013) Monocyte and macrophage heterogeneity in the heart. Circ Res 112:1624–1633 23743228 10.1161/CIRCRESAHA.113.300890 PMC3753681

[CR32] Pandi A, Raghu MH, Chandrashekar N, Kalappan VM (2022) Cardioprotective effects of ferulic acid against various drugs and toxic agents. Beni Suef Univ J Basic Appl Sci 11:92

[CR33] Porzionato A, Rucinski M, Macchi V, Stecco C, Malendowicz LK, De Caro R (2010) Spexin expression in normal rat tissues. J Histochem Cytochem 58:825–837 20530460 10.1369/jhc.2010.956300 PMC2924798

[CR34] Porzionato A, Rucinski M, Macchi V, Stecco C, Sarasin G, Sfriso MM, Di Giulio C, Malendowicz LK, De Caro R (2012) Spexin is expressed in the carotid body and is upregulated by postnatal hyperoxia exposure. Adv Exp Med Biol 758:207–213 23080164 10.1007/978-94-007-4584-1_29

[CR35] Ray PD, Huang BW, Tsuji Y (2012) Reactive oxygen species (ROS) homeostasis and redox regulation in cellular signaling. Cell Signal 24:981–990 22286106 10.1016/j.cellsig.2012.01.008 PMC3454471

[CR36] Říha M, Vopršalová M, Pilařová V, Semecký V, Holečková M, Vávrová J et al (2014) Oral administration of quercetin is unable to protect against isoproterenol cardiotoxicity. Naunyn Schmiedebergs Arch Pharmacol 387:823–835 24899384 10.1007/s00210-014-0995-z

[CR37] Sasaki T, Shimazawa M, Kanamori H, Yamada Y, Nishinaka A, Kuse Y et al (2020) Effects of progranulin on the pathological conditions in experimental myocardial infarction model. Sci Rep 10:11842 32678228 10.1038/s41598-020-68804-7 PMC7367277

[CR38] Sasaki T, Kuse Y, Nakamura S, Shimazawa M, Hara H (2023) Progranulin deficiency exacerbates cardiac remodeling after myocardial infarction. FASEB Bioadv 5:395–411 37810172 10.1096/fba.2023-00084 PMC10551273

[CR39] Schmitz K, Wilken-Schmitz A, Vasic V, Brunkhorst R, Schmidt M, Tegeder I (2020) Progranulin deficiency confers resistance to autoimmune encephalomyelitis in mice. Cell Mol Immunol 17:1077–1091 31467413 10.1038/s41423-019-0274-5 PMC7609649

[CR40] Senoner T, Dichtl W (2019) Oxidative stress in cardiovascular diseases: still a therapeutic target? Nutrients 11:2090 31487802 10.3390/nu11092090 PMC6769522

[CR41] Shanab O, El-Rayes SM, Khalil WF, Ahmed N, Abdelkader A, Aborayah NH et al (2023) Nephroprotective effects of *Acacia senegal* against aflatoxicosis via targeting inflammatory and apoptotic signaling pathways. Ecotoxicol Environ Saf 262:115194 37385018 10.1016/j.ecoenv.2023.115194

[CR42] Shanab O, Mostafa L, Abdeen A, Atia R, Nassar AY, Youssef M et al (2024) Modulatory mechanisms of copper(II)-albumin complex toward N-nitrosodiethylamine-induced neurotoxicity in mice via regulating oxidative damage, inflammatory, and apoptotic signaling pathways. Ecotoxicol Environ Saf 270:115841 38113799 10.1016/j.ecoenv.2023.115841

[CR43] Song L, Srilakshmi M, Wu Y, Saleem TSM (2020) Sulforaphane attenuates isoproterenol-induced myocardial injury in mice. BioMed Res Int 2020:3610285 33415146 10.1155/2020/3610285 PMC7769644

[CR44] Studneva I, Palkeeva M, Veselova O, Molokoedov A, Ovchinnikov M, Sidorova M et al (2019) Protective effects of a novel agonist of galanin receptors against doxorubicin-induced cardiotoxicity in rats. Cardiovasc Toxicol 19:136–146 30238355 10.1007/s12012-018-9483-x

[CR45] Sun M, Yin F, Wu X, Sun S, An Y, Zhu M et al (2024) Effects of ivabradine on myocardial autophagy and apoptosis in isoprenaline-induced heart failure in mice. Iran J Basic Med Sci 27:107–113 38164488 10.22038/IJBMS.2023.70060.15236 PMC10722486

[CR46] Tao L, Wang L, Yang X, Jiang X, Hua F (2019) Recombinant human glucagon-like peptide-1 protects against chronic intermittent hypoxia by improving myocardial energy metabolism and mitochondrial biogenesis. Mol Cell Endocrinol 481:95–103 30503377 10.1016/j.mce.2018.11.015

[CR47] Tian R, Li Y, Yao X (2016) PGRN suppresses inflammation and promotes autophagy in keratinocytes through the Wnt/β-catenin signaling pathway. Inflammation 39:1387–1394 27239673 10.1007/s10753-016-0370-y

[CR48] Wei YJ, Wang JF, Cheng F, Xu HJ, Chen JJ, Xiong J, Wang J (2021) miR-124-3p targeted SIRT1 to regulate cell apoptosis, inflammatory response, and oxidative stress in acute myocardial infarction in rats via modulation of the FGF21/CREB/PGC1α pathway. J Physiol Biochem 77:577–587 34146302 10.1007/s13105-021-00822-z

[CR49] Wu J, Cui Y, Ding W, Zhang J, Wang L (2024) The protective effect of macrostemonoside T from *Allium macrostemon* Bunge against isoproterenol-induced myocardial injury via the PI3K/Akt/mTOR signaling pathway. Int Immunopharmacol 133:112086 38642441 10.1016/j.intimp.2024.112086

[CR50] Yalçin T, Kaya S, Yiğin A, Ağca CA, Özdemir D, Kuloğlu T, Boydak M (2024) The effect of thymoquinone on the TNF-α/OTULIN/NF-κB axis against cisplatin-induced testicular tissue damage. Reprod Sci 31:2433–2446 38658488 10.1007/s43032-024-01567-y PMC11289327

[CR51] Yalçin T, Kaya S (2025) Effect of thymoquinone on hippocampal spexin levels in cisplatin-induced rats. Neurol Res 47(9):817–825 40340641 10.1080/01616412.2025.2504158

[CR52] Yin Y, Wang L, Chen G, You H (2022) Effect of fraxetin on oxidative damage caused by isoproterenol-induced myocardial infarction in rats. Appl Biochem Biotechnol 194:5666–5679 35802243 10.1007/s12010-022-04019-y

[CR53] Yue Y, Zhao H, Yue Y, Zhang Y, Wei W (2020) Downregulation of microRNA-421 relieves cerebral ischemia/reperfusion injuries: involvement of anti-apoptotic and antioxidant activities. Neuromol Med 22:411–419 10.1007/s12017-020-08600-8 32385800

[CR54] Zarkovic N (2020) Roles and functions of ROS and RNS in cellular physiology and pathology. Cells 9:767 32245147 10.3390/cells9030767 PMC7140712

[CR55] Zhou M, Tang W, Fu Y et al (2015) Progranulin protects against renal ischemia/reperfusion injury in mice. Kidney Int 87:918–929 25607110 10.1038/ki.2014.403

[CR56] Zhou T, Chen Y, Zhang S, Li M, Wang J (2021) Serum progranulin as a risk predictor in patients with acute myocardial infarction. Med Sci Monit 27:e928864 33635854 10.12659/MSM.928864 PMC7923397

[CR57] Zhuang L, Kong Y, Yang S, Lu F, Gong Z, Zhan S, Liu M (2021) Dynamic changes of inflammation and apoptosis in cerebral ischemia-reperfusion injury in mice investigated by ferumoxytol-enhanced magnetic resonance imaging. Mol Med Rep 23:282 33604682 10.3892/mmr.2021.11921 PMC7905325

